# Transcriptome analysis reveals regulation mechanism of methyl jasmonate-induced terpenes biosynthesis in *Curcuma wenyujin*

**DOI:** 10.1371/journal.pone.0270309

**Published:** 2022-06-23

**Authors:** Qiuhui Wei, Kaer Lan, Yuyang Liu, Rong Chen, Tianyuan Hu, Shujuan Zhao, Xiaopu Yin, Tian Xie

**Affiliations:** 1 School of Pharmacy, Hangzhou Normal University, Hangzhou, Zhejiang, China; 2 Key Laboratory of Elemene Class Anti-Cancer Chinese Medicines, Engineering Laboratory of Development and Application of Traditional Chinese Medicines, Collaborative Innovation Center of Traditional Chinese Medicines of Zhejiang Province, Hangzhou Normal University, Hangzhou, Zhejiang, China; Huazhong University of Science and Technology, CHINA

## Abstract

*Curcuma wenyujin* is the source plant of three traditional Chinese medicines, which have been widely used in clinical treatment over 1000 years. The content of terpenes, the major medicinal active ingredients, is relatively low in this plant. Studies have shown that MeJA can promote terpenes biosynthesis in plants. However, the mechanism underlying the effect of MeJA in *C*. *wenyujin* remains unclear. In this work, the transcriptome of *C*. *wenyujin* leaves with MeJA treatment was analyzed to elucidate the regulation mechanism of MeJA-mediated terpene biosynthesis. Based on the RNA-seq data, 7,246 unigenes were differentially expressed with MeJA treatment. Expression pattern clustering of DEGs revealed that unigenes, related to JA biosynthesis and signal transduction, responded to exogenous MeJA stimulation on the early stage and maintained throughout the process. Subsequently, unigenes related to terpene biosynthesis pathway showed a significant up-regulation with 6 h treatment. The analysis results suggested that MeJA induced the expression of JA biosynthesis genes (such as *LOXs*, *AOSs*, *AOCs*, *OPRs*, and *MFPs*) and JA signal transduction core genes (*JAZs* and *MYCs*) to activate JA signaling pathway. Meanwhile, downstream JA-responsive genes presented up-regulated expression levels such as *AACT*, *HMGSs*, *HMGRs*, *DXSs*, *DXRs*, *MCTs*, *HDSs*, and *HDRs*, thus promoting terpenes biosynthesis. The transcriptional expressions of these genes were validated by qRT-PCR. In addition, six *CwTPS* genes in response to MeJA were identified. With MeJA treatment, the expression levels of *CwTPSs* were increased as well as those of the transcription factors MYB, NAC, bZIP, WRKY, AP2/ERF, and HLH. These TFs might potentially regulate terpenes biosynthesis. These results provide insights for regulation mechanism of terpenes biosynthesis.

## Introduction

*Curcuma wenyujin* Y. H. Chen et C. Ling (*C*. *wenyujin*) is a member of *Zingiberaceae* family, which is distributed in tropical/subtropical regions and primarily cultivated in Zhejiang Province, P. R. China. As the source plant of three traditional Chinese medicines pian-jiang-huang, wen-e-zhu, and wen-yu-jin, *C*. *wenyujin* has been widely used in clinical treatment because of the various pharmacological activities such as anti-tumor, anti-inflammatory, antioxidant, antimicrobial and antiviral activities [1, 2]. In the past decades, the extensive application of *C*. *wenyujin* has stimulated a rapid increase in research on its active ingredients, especially in essential oil extracted from *C*. *wenyujin* rhizome. A total of 170 compounds containing 125 sesquiterpenes, 7 monoterpenes, 6 diterpenes, 18 curcuminoids, and 14 others have been identified in *C*. *wenyujin* [1, 3]. However, the content of terpenes, the major medicinal active ingredients, is relatively low in this plant. The production of plant-derived terpenes is barely to meet the increasing demand in the pharmaceutical industry. Due to the limited understanding of complex metabolic pathway, the heterologous production of terpenes in microbial hosts is dissatisfied at present. Therefore, revealing the regulation mechanism has become one of the most important subjects to increase the accumulation of terpenes in *C*. *wenyujin*.

Plant terpenes derive from the same five-carbon intermediate products isopentyl diphosphate (IPP) and its double-bond isomer dimethylallyl diphosphate (DMAPP), which are synthesized via 2-C-methyl-D-erythritol 4-phosphate (MEP) and mevalonate (MVA) pathways. Then, the five-carbon intermediates combine with each other to form long chain prenyl diphosphate molecules (geranyl diphosphate, GPP; farnesyl diphosphate, FPP; or geranylgeranyl diphosphate, GGPP), which are precursors for terpenes synthesis [4, 5]. Although the MVA pathway and MEP pathway operate in cytosol and plastid respectively, they are not totally independent of each other. Their interaction depends on intermediates (such as IPP, GPP, FPP, and GGPP) exchange [6]. The extent of the cross-talk between MEP and MVA pathways strongly depends on internal and external stimuli [7]. Furthermore, terpene synthases (TPS) are the key enzymes in terpene biosynthesis, which can produce a variety of terpene products such as isoprene, monoterpenes, sesquiterpenes, diterpenes, etc [8, 9]. The functional diversity of TPSs results in the abundant terpene products in plants. The production and accumulation of terpene metabolites are the main mechanism for plants to resist adverse environment [10]. The regulation of terpenes biosynthesis occurs at multiple levels, including transcriptional and post-translational levels [4, 11].

Hormones play important roles in regulating plant growth and development. Jasmonic acid (JA) and its derivative methy jasmonate (MeJA) have been reported to modulate the accumulation of many secondary metabolites, such as anthocyanins, nicotine, terpenoid indole alkaloids, glucosinolates and artemisinin [12]. For example, MeJA induced phenolic acids and tanshinones biosynthesis as an exogenous elicitor in *Salvia miltiorrhiza* hairy root [13, 14] as well as flavonoid biosynthesis in different fruit species [15, 16]. Terpene biosynthesis was also regulated by exogenous MeJA elicitor. High-throughput sequencing technologies have been widely used to clarify the regulation mechanism of MeJA in plant terpene biosynthesis. In *Sindora glabra*, external application of MeJA significantly led to different responses of genes involved in the MVA and MEP pathways. Most genes of MVA pathway exhibited minimal expression with exception of the rate-limiting enzyme genes *HMGR1* and *HMGR2* which exhibited increased expression levels. In contrast, the majority of genes in the MEP pathways were dramatically down-regulated. The accumulation of two sesquiterpenes α-copaene and β-caryophyllene, was increased under the induction of MeJA in *S*. *glabra* [17]. In *Pogostemon cablinafter*, MeJA treatment significantly increased the content of patchouli alcohol, a sesquiterpene compound, which is the main component in patchouli oil. Most of the genes in the MVA and MEP pathways were up-regulated with MeJA treatment, in accord with the increasing tendency of patchoulol content [18]. Although the content of sesquiterpenes was increased in *S*. *glabra* and *P*. *cablinafter* respectively, the response patterns of structure genes of MEP and MVA pathways to MeJA were different. It seems that different plants have different mechanisms of terpene biosynthesis with MeJA treatment. In addition, transcription factors (TFs) were co-expressed with terpene biosynthesis genes under MeJA induction, including MYBs (v-myb avian myeloblastosis viral oncogene homologs), AP2/ERFs (apetala2/ethylene-responsive factors), WRKYs, NACs (NAM, ATAF, and CUC), and bHLH (basic/helix-loop-helix) etc. These TFs might potentially regulate terpene biosynthesis. For example, in *Artemisia annua*, JA treatment induced degradation of AaJAZ8. As the result, the transcriptional complex AaTCP14-AaORA subsequently activated the expression of *DBR2*, which was essential for artemisinin biosynthesis [19]. In *Betula platyphylla*, the expression levels of *BpMYB21* and *BpMYB61* were induced by MeJA. Overexpression of *BpMYB21* and *BpMYB61* in *B*. *platyphylla* affected the content of triterpenoids including betulinic acid, oleanolic acid, and botulin, through activating squalene epoxidase (*SE*) and b-amyrin synthase (*BPX*) genes of triterpene biosynthesis pathway [20]. The regulation mechanisms between TFs and function genes are different and diverse in different plants.

Sesquiterpenes are the main terpenes in essential oil extracted from *C*. *wenyujin*. Genomic resources and transcriptome sequences for *C*. *wenyujin* are very limited at present. To date, regulation of MeJA-induced terpenes biosynthesis in *C*. *wenyujin* is completely unknown. In this study, the comparative transcriptome analysis was performed to examine differences in genes expression between the MeJA-treated and untreated *C*. *wenyujin*. Moreover, transcriptomic analysis also can be used to identify functional elements in metabolic pathways or regulation networks. In this study, a series of candidate genes were identified to be involved in terpenes biosynthesis and transcriptional regulation in responding to JA signal. Co-expression analysis was used to evaluate the potential regulation relationships between TFs and function genes. These candidate genes as well as detailed regulation mechanisms will be characterized in the further study.

## Materials and methods

### Plant materials and treatment

*C*. *wenyujin* was used as the plant material in this study. The germplasm was obtained from Wenzhou City, Zhejiang province, P.R. China (14 m altitude, 27°47’N, 120°37’E). The original plants and its macroscopic characters were authenticated by Professor Zengxi Guo who works in the institute of food and drug control in Zhejiang, P.R. China. Briefly, buds of seed tubers of *C*. *wenyujin* were sterilized and cultured in Murashige and Skoog (MS) solid medium supplemented with 30 g/L sucrose and 3 mg/L 6-benzylaminopurine (6-BA) to induce tufted seedlings. After one month, the small tufted seedlings were divided into single plant and cultured to trefoil stage in MS solid medium. Then, seedlings with similar growth were collected and treated in MS liquid medium with 250 μM MeJA for 1 h or 6 h, respectively. The culture bottles with caps were used to prevent the emission of volatile hormone and for more absorbtion of elicitor. The seedlings were cultured in MS liquid medium without MeJA as control. Each group contained six seedlings as replicates. The culture and treatment of seedlings were performed in the incubator with 12 h light/12 h dark cycle at 22°C. Leaves samples of *C*. *wenyujin* were collected at 0, 1, and 6 h after MeJA induction. At each time-point, samples were collected from six seedlings (six replicates), frozen in liquid nitrogen, and then stored at -80°C for subsequent RNA extraction. For content analysis, the seedlings were transferred from MS medium to soil and cultured in greenhouse for one month under 12 h light/12 h dark cycle at 25°C. Then, the seedlings were sprayed with 250 μM MeJA every 24 hours for 8 days. The leaves were collected and stored at -80°C for subsequent content determination.

### RNA extraction and sequencing

Three of six replicate samples were selected randomly for RNA sequencing (RNA-seq). Total RNA was extracted from the collected samples with the RNA Extraction Kit (MJYH, shanghai, China). RNA quality was determined by 2100 Bioanalyser (Agilent, CA, USA) and quantified using the NanoDrop 2000 (Thermo, Wilmington, DE, USA). The high-quality RNA sample (OD260/280 = 1.8–2.2, OD260/230 ≥ 1.0, RIN ≥ 8) was used to construct a sequencing library. RNA-seq library was prepared following guide of Illumina TruseqTM RNA sample prep Kit (Illumina Inc., San Diego, USA) using 1 μg of total RNA. After quantified by TBS380 Fluorometer (Turner BioSystems, USA), paired-end library was sequenced on the Illumina NovaSeq6000 platform (Majorbio Biological Pharmaceutical Co., Ltd., Shanghai, China).

## *De novo* transcriptome assembly and transcriptomic analysis

The raw data was quality controlled by fastp software (https://github.com/OpenGene/fastp). After removing adapter sequences, the reads with ambiguous bases ‘N’, and trimming the low-quality bases in 3’-end of reads, the clean reads (length ≥ 30 bp) were acquired for *de novo* assembly. *De novo* assembly was performed using Trinity (https://github.com/trinityrnaseq/trinityrnaseq/wiki), optimized using TransRate (http://hibberdlab.com/transrate/), and removed redundancy using CD-HIT (http://weizhongli-lab.org/cd-hit/). Then non-redundant transcripts and unigenes were combined to give a reference transcriptome. Original RNA-sequence data was deposited in National Genomics Data Center (https://ngdc.cncb.ac.cn/) with the accession number CRA006461.

All the unigenes and transcripts were annotated in the databases of NR (the NCBI non-redundant, protein sequences) [21], SWISS-PROT (a manually annotated and reviewed protein sequence database) [22], PFAM (Protein family) [23], COG (Cluster of Orthologous Groups of proteins) [24], GO (Gene Onthology) [25] and KEGG (Kyoto Encyclopedia of Genes and Genomes) [26] to explore their possible functions. The CDS prediction was performed by aligning all unigenes and transcripts with NR/Swissprot protein database. The CDSs unmapped or without predicted sequences in protein database were predicted by Trans-Decoder (https://github.com/TransDecoder/). The expression level of each transcript was quantified using the RSEM program [27] and present with transcripts per million reads (TPM) values. Differentially expressed genes (DEGs) in samples were identified using DESeq2 software [28] with p-adjust < 0.05 and |log2 Fold Chang| ≥ 1. Expression pattern clustering of DEGs was performed using Short Time-series Expression Miner (STEM) software with *p* < 0.05 [29]. The GO functional enrichment and KEGG pathway enrichment analysis were conducted using the Goatools (https://github.com/tanghaibao/Goatools) [30] and KOBAS (http://kobas.cbi.pku.edu.cn/) with corrected *p*-value < 0.05.

Heatmaps were generated from log2 based (TPM) values using TB tools heatmap illustrator [31]. TPM of these unigenes were obtained from this RNA-seq data. The expression correlation analysis was performed using spearman method with corrected *p*-value < 0.05 and correlation coefficient threshold >0.9. Networks were visualized using Cytoscape software [32].

### Identification and evolutionary analysis of the putative CwTPSs

Terpene_synth domain HMM profiles (PF01397, PF03936, and PF19086) were downloaded from pfam database. Then terpene_synth domain HMM profiles were searched in all DEGs of *C*. *wenyujin* transcriptome. These unigenes were also queried into NR database to detect the homologous sequences. Multiple sequence alignments of CwTPSs were performed using ClustalW software with the default parameters. The phylogenetic tree was generated using the neighbor-joining (NJ) method with 1000 bootstrap replicates in MEGA 11 [33].

### Quantitative RT-PCR analysis

Samples collected for transcriptome analysis were also used for determination of genes expression. First-strand cDNA was synthesized with the FastQuant RT Kit (TaKaRa, Dalian, China). qRT-PCR was performed with SuperReal PreMix Plus Kits (Zoman, Beijing, China) utilizing the CFX Connect Real-Time System (Bio-Rad, Hercules, CA, United States). Expression data was analyzed with the 2^−ΔΔCt^ method [34]. Statistical analysis was performed using SPSS software (IBM, New York, US). Statistical significance was set as *p < 0.05 and **p < 0.01. The housekeeping gene of 18S rRNA was used as the internal control. The primers used in this assay were listed in S1 Table.

### β-elemene extraction and analysis

The collected leaf samples were dried at a vacuum freeze drier. 0.15 g of dried leaf powder was soaked in 10 ml n-hexane for 12 h, then, extracted with 40,000 Hz ultrasonic for 60 min at room temperature. The supernatant was collected after centrifugation and passed through a 0.22-μm organic membrane. The extraction was analyzed using gas chromatograph (GC, Shimadzu GC-2010 plus) with an Agilent DB-225 column (30 m × 250 mm × 0.25 mm film thickness). The injection volume was 1 μl of continuous filtrate, and nitrogen was the carrier gas. The GC temperature program was as follows: the initial temperature 80°C, increased to 140°C at a rate of 5°C/min, raised to 220°C at a rate of 10°C/min for 2 min. Flame-ionization detector (FID) temperature was 305°C and injection port temperature was 260°C. The external standard method was used for quantification. The β-elemene standard was purchased from National Institutes for Food and Drug Control (Beijing, China). Three biological replicates and two technical replicates were performed.

## Results

### Transcriptome sequencing and assembly

Total RNA was extracted from MeJA-treated and untreated samples of *C*. *wenyujin* for RNA-seq analysis. A total of 82.43 Gb clean data (85.62 Gb raw data) were generated, of which over 6.57 Gb clean data for each sample. The Q20 and Q30 percentages were higher than 97% and 93%, respectively. The average GC content of the transcriptome was 50.05%. Mapping the clean reads to the reference sequences assembled by Trinity showed that the mapped ratio ranged from 80.57% to 83.07% (S2 Table). The transcriptome was assembled to 98,647 unigenes, with an N50 = 1,574 bp. The smallest and largest length of unigenes was 201 bp and 15,577 bp, respectively. In expression analysis, 97,895 unigenes and 184,965 transcripts were detected, respectively.

### Expression pattern clustering of DEGs

**Fig 1:**
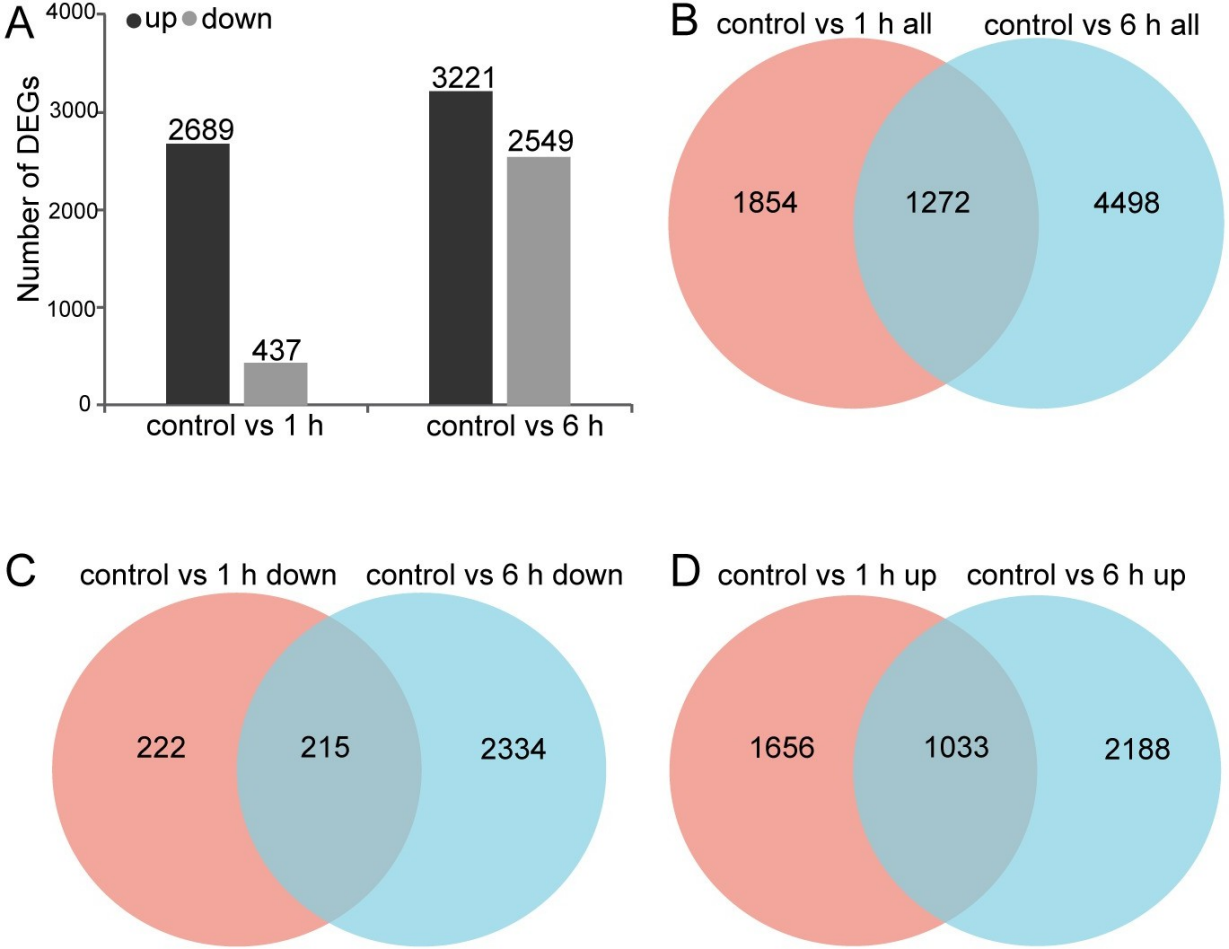
Comparative statistics of differentially expressed genes (DEGs) in MeJA-treated *C*. *wenyujin*. (A) Total number of up-regulated and down regulated genes in different groups (group 1: control vs 1 h; group 2: control vs 6 h). (B-D) Venn diagram illustrating the shared and unique DEGs between different groups. The overlaps indicate the number of shared DEGs between different groups.

DEGs were identified and filtered in MeJA-treated *C*.*wenyujin* according to the following criteria: adjusted *p* value < 0.05 and fold change > 2. A total of 7,246 DEGs were detected. In control vs 1 h group, the expression levels of 2,689 DEGs were up-regulated, while those of 437 were down-regulated. After 6 h treatment, the expression levels were up-regulated for 3,221 DEGs and down-regulated for 2,549 DEGs. Among them, 1,033 up-regulated and 215 down-regulated DEGs were detected in both groups “control vs 1 h” and “control vs 6 h” (Fig 1 and S1 Fig).

**Fig 2:**
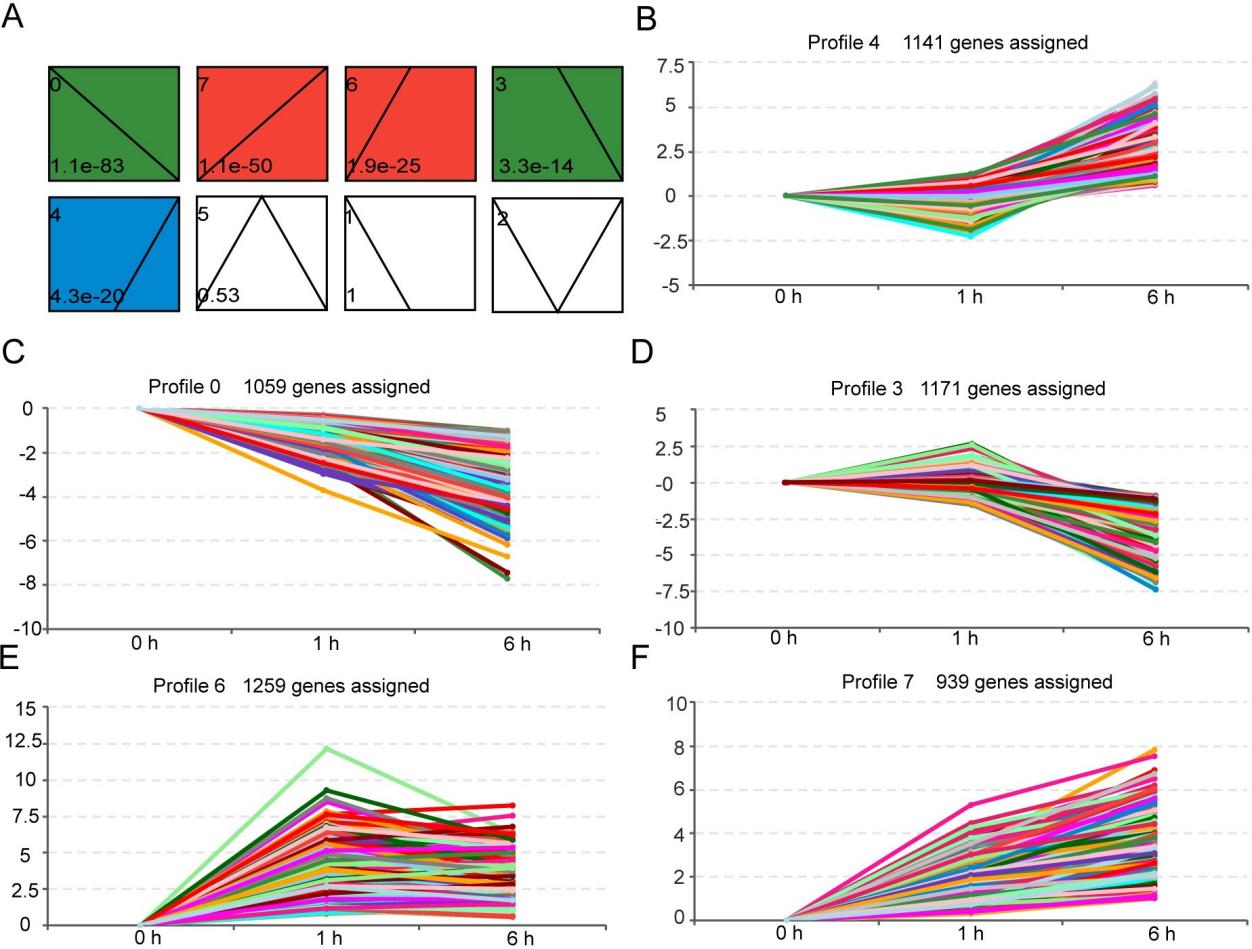
Patterns of gene expression across three time points in *C*. *wenyujin* with MeJA treatment. (A) Expression trend clustering of all DEGs. Each rectangle represents a profile. Profiles with the same color can be grouped into the same cluster. The colorless rectangle indicates that clustering is not significant. The number in the upper left corner of the rectangle is the number of profile, in which the broken line is the trend of expression over time. The *p*-value in the lower left corner reflects the corresponding significance level. (B-F) Expression patterns of each significant clustering profile. The number of unigenes in each profile is labeled above the frame. Broken lines represent the trend of gene expression. The numbers on the x-axis indicate the time points of 0, 1, and 6 h, respectively. The numbers on the y-axis represent up-regulation (> 0), no change (= 0), and down-regulation (< 0), respectively.

To gain further insight into the time course of unigenes expression with MeJA treatment, the trend analysis of all DEGs was conducted. According to the gene expression patterns, DEGs were clustered into 8 profiles with the software STEM. Five of these profiles exhibited significant clustering of unigene expression patterns with MeJA treatment (*p* < 0.05) (Fig 2A). DEGs were clustered to profiles 0 and 7, presenting transcriptional down-regulation or up-regulation during the whole process of MeJA treatment (Fig 2C and 2F). DEGs clustered to profiles 6 presented transcriptional changes, which occurred at 1 h after MeJA induction and remained till the end of treatment (Fig 2E). DEGs clustered to profiles 3 and 4, presented transcriptional changes that only occurred 6 h after MeJA induction (Fig 2B and 2D). The GO enrichment analysis for each profile showed that DEGs clustered in profile 6 and 4 were significantly enriched in JA mediated signaling pathway and terpenes biosynthesis pathway, respectively (S2 Fig). The results revealed that unigenes involved in MeJA-induced terpenes biosynthesis might be hidden in these two profiles.

### GO and KEGG enrichment analysis

**Fig 3:**
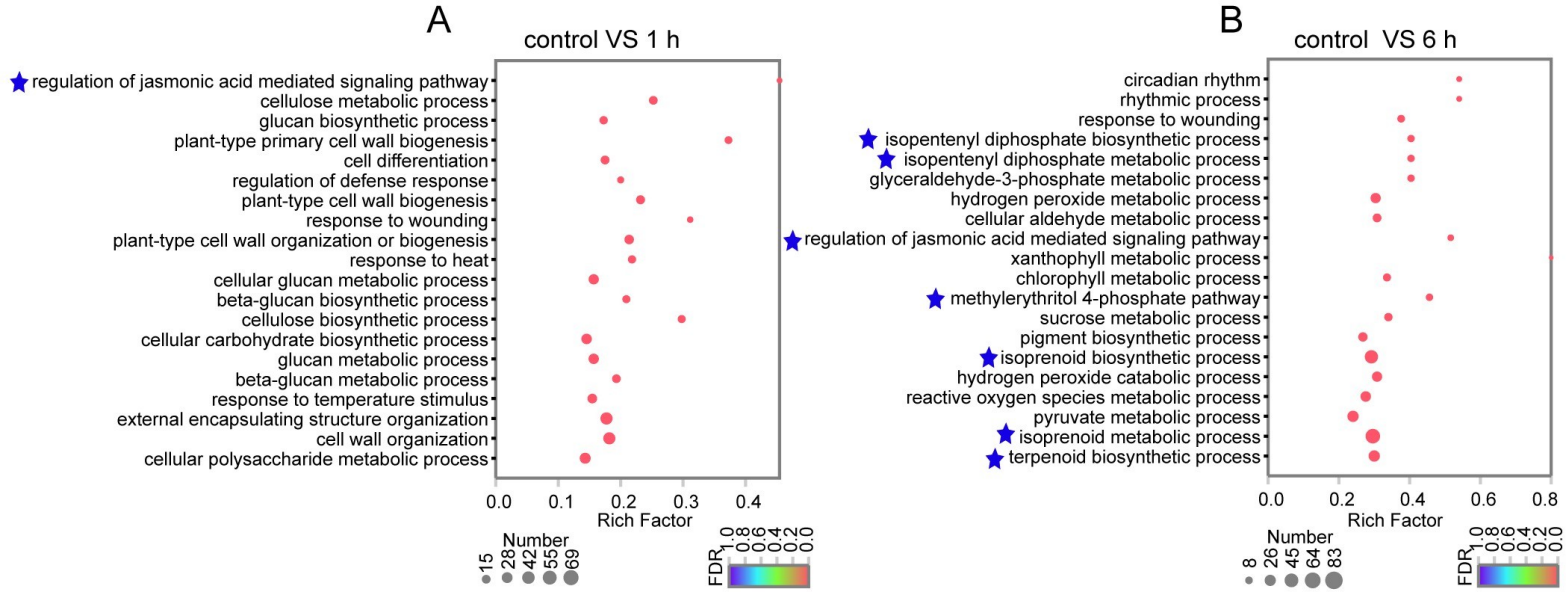
GO enrichment analysis of DEGs in *C*. *wenyujin* with MeJA-treatment for “control vs 1 h” (A) and “control vs 6 h” (B) groups. The y-axis on the right side represents the GO terms and x-axis represents rich factor. The size of the dot indicates the number of genes in this GO term, and the color of the dot corresponds to different FDR (*p*-vaule). *p*-vaule < 0.05 indicates significant enrichment of GO function. Top 20 of significant enrichment results are displayed.

The results of GO classification showed that 3,126 DEGs in “control vs 1 h” were grouped into 43 GO terms, and 5,770 DEGs in “control vs 6 h” were grouped into 47 GO terms (S3 Fig). Then GO enrichment analysis for all DEGs was performed with adjusted *p* < 0.05. DEGs in *C*. *wenyujin* treated with MeJA for 1 h and 6 h were significantly enriched in “regulation of jasmonic acid mediated signaling pathway” (GO:2000022). In addition, DEGs in *C*. *wenyujin* with 6 h MeJA-treatment were significantly enriched in terpene biosynthesis related processes (GO:0009240, GO:0019288, GO:0046490, GO:0008299, GO:0006720, and GO:0016114) (Fig 3).

**Fig 4:**
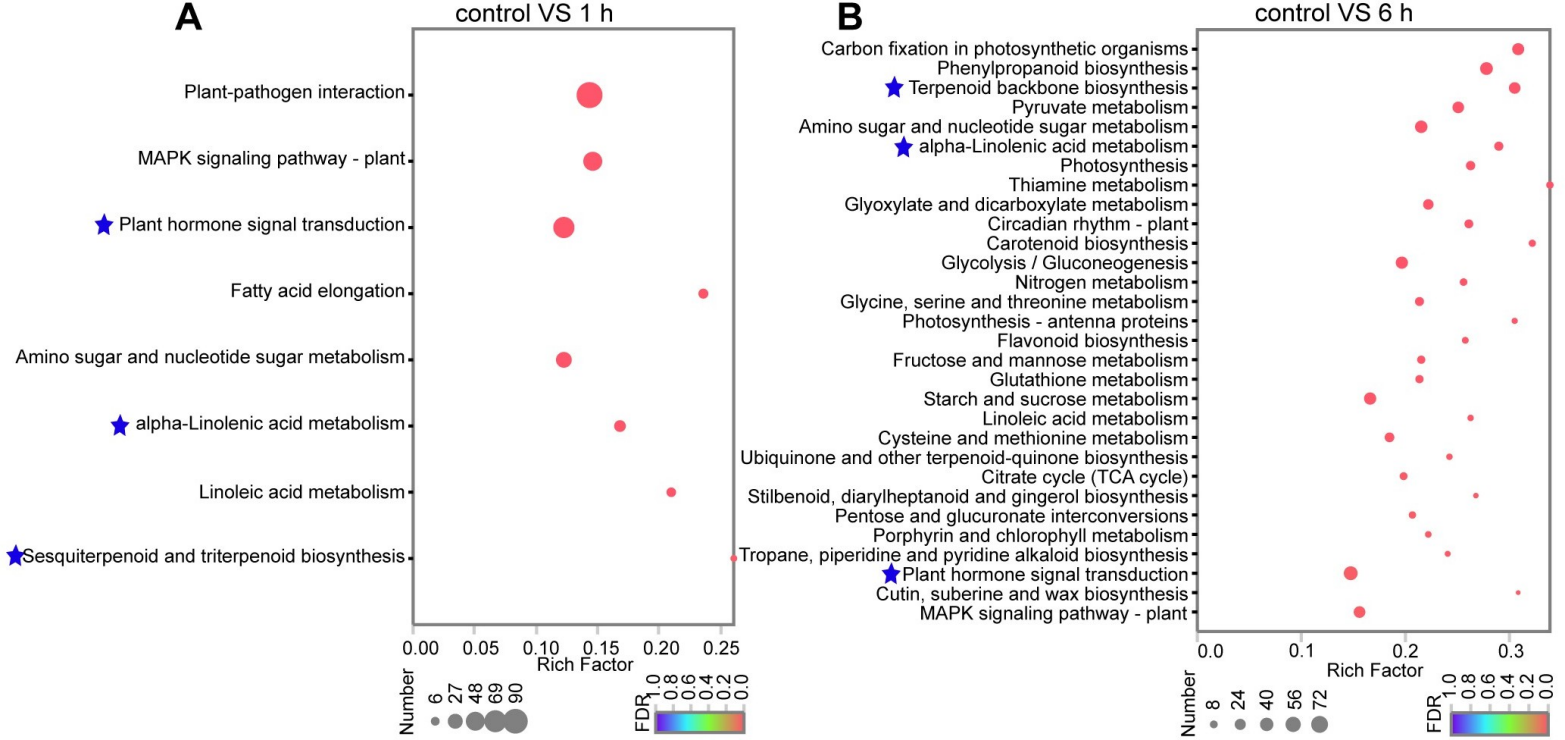
KEGG enrichment analysis of DEGs in *C*. *wenyujin* with MeJA-treatment for “control vs 1 h” (A) and “control vs 6 h” (B) groups. The y-axis on the right side represents the KEGG pathway and x-axis represents rich factor. The size of the dot indicates the number of genes in KEGG pathway, and the color of the dot corresponds to different FDR (*p*-vaule). *p*-vaule < 0.05 indicates significant enrichment of KEGG pathway. Up to top 30 of significant enrichment results are displayed.

The KEGG enrichment analysis was also performed with adjusted *p* < 0.05 to elucidate the biological pathways activated by MeJA treatment. DEGs in *C*. *wenyujin* with 1 h and 6 h MeJA-treatment were both significantly enriched in “plant hormone signal transduction” (map04075), “alpha-Linolenic acid metabolism” (map00592), “terpenoid backbone biosynthesis” (map00900), “sesquiterpenoid and triterpenoid biosynthesis” (map00909) (Fig 4). All GO terms and KEGG pathways mentioned above were associated with JA biosynthesis, JA signal transduction, and terpene biosynthesis.

### Analysis of DEGs related to terpene backbone biosynthesis pathway

**Fig 5:**
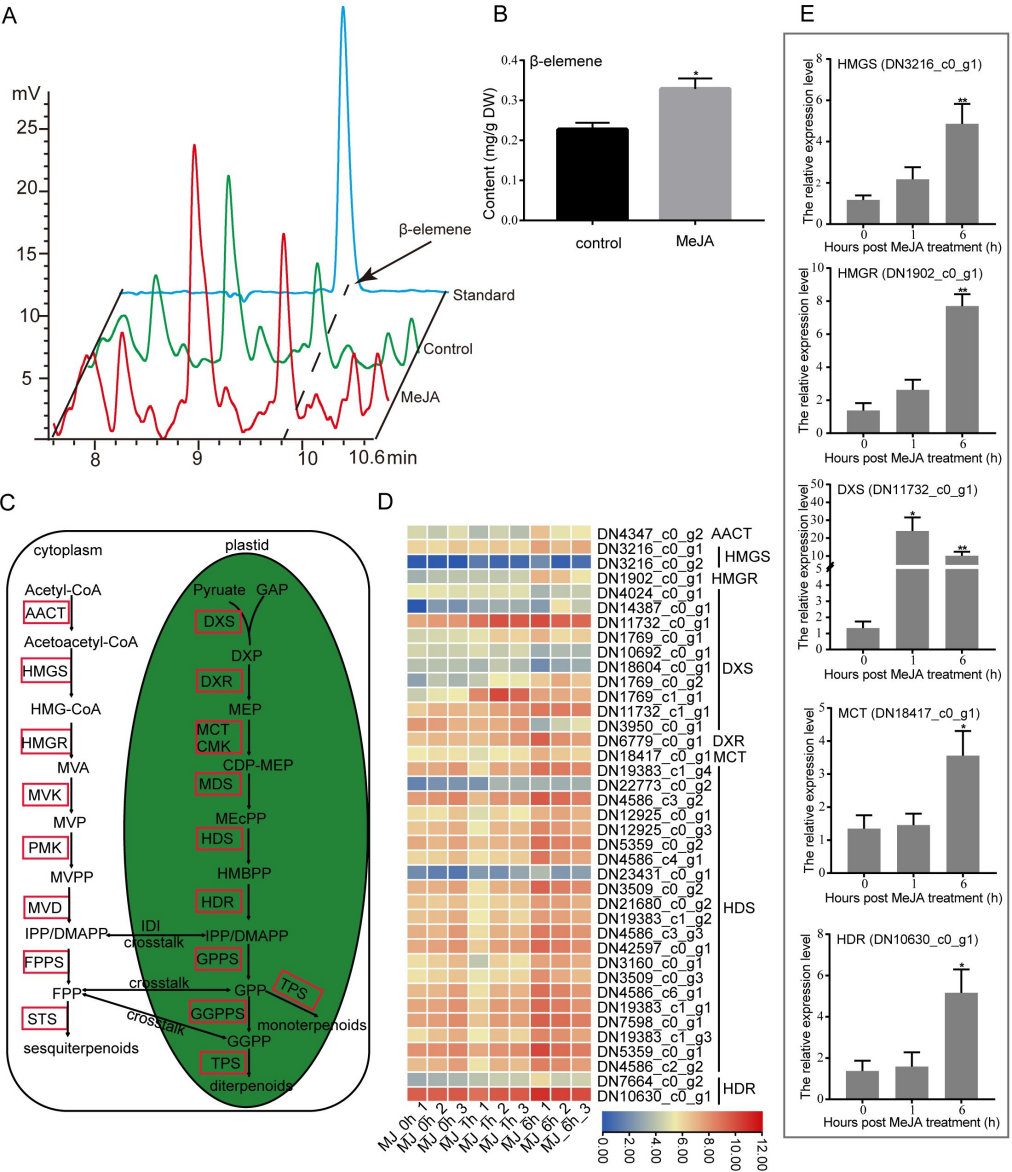
Analysis of DEGs related to terpene backbone biosynthesis pathway in *C*. *wenyujin* with MeJA-treatment. (A) Gas chromatogram of leaves extracts from plant sprayed with or without MeJA. (B) Contents of β-elemene in leaves. (C) The terpene backbone biosynthesis pathway. Genes with red box represent enzyme genes in this biosynthesis pathway (D) Transcriptional profiles of DEGs related to terpene backbone biosynthesis pathway. The log2 (TPM) values for the DEGs were calculated based on three biological replicates for each time-point. The color scale from blue to red represents increase expression level. (E) Expression level of DEGs determined by qRT-PCR. Three independent biological replicates were performed. Asterisks indicate significant difference (*p< 0.05; **p < 0.01).

In this study, the content of β-elemene, the main active ingredient of *C*.*wenyujin*, was significantly increased after MeJA treatment (Fig 5A and 5B). We further detected the expression patterns of genes related to β-elemene biosynthesis in response to MeJA treatment. The isoprenoid precursors for terpenes are synthesized through the crosstalk pathway MEP and MVA in plants. In both pathways, thirty-nine structural genes with significantly differential expression levels were identified (S3 Table). For MEP pathway in plastids, DXP synthase (*DXS*), reductoisomerase (*DXR*), MEP cytidyltransferase (*MCT*), HMBPP synthase (*HDS*), and reductase (*HDR*) genes were differentially expressed with MeJA treatment. These DEGs encoded a series of enzymes to catalyze glyceraldehydyde-3-phosphate (GAP) and pyruvate to synthetize intermediate products IPP and DMAPP. In addition, four DEGs encoding acetoacetyl-CoA thiolase (AACT), HMG-CoA synthase (HMGS) and reductase (HMGR) in MVA pathway were identified with MeJA treatment. These identified structural genes were all mapped in terpene biosynthesis pathway, meanwhile expression patterns of them were also determined (Fig 5C and 5D). The expression levels of all identified structure genes were significantly up-regulated in the MeJA-treated *C*. *wenyujin* especially with 6 h treatment, excepting that four *DXS* genes were down-regulated. The reliability of RNA-seq data were confirmed by further determination of the relative expression levels of selected structural genes with method of qRT-PCR (Fig 5E).

### Analysis of DEGs related to JA biosynthesis and signal transduction

**Fig 6:**
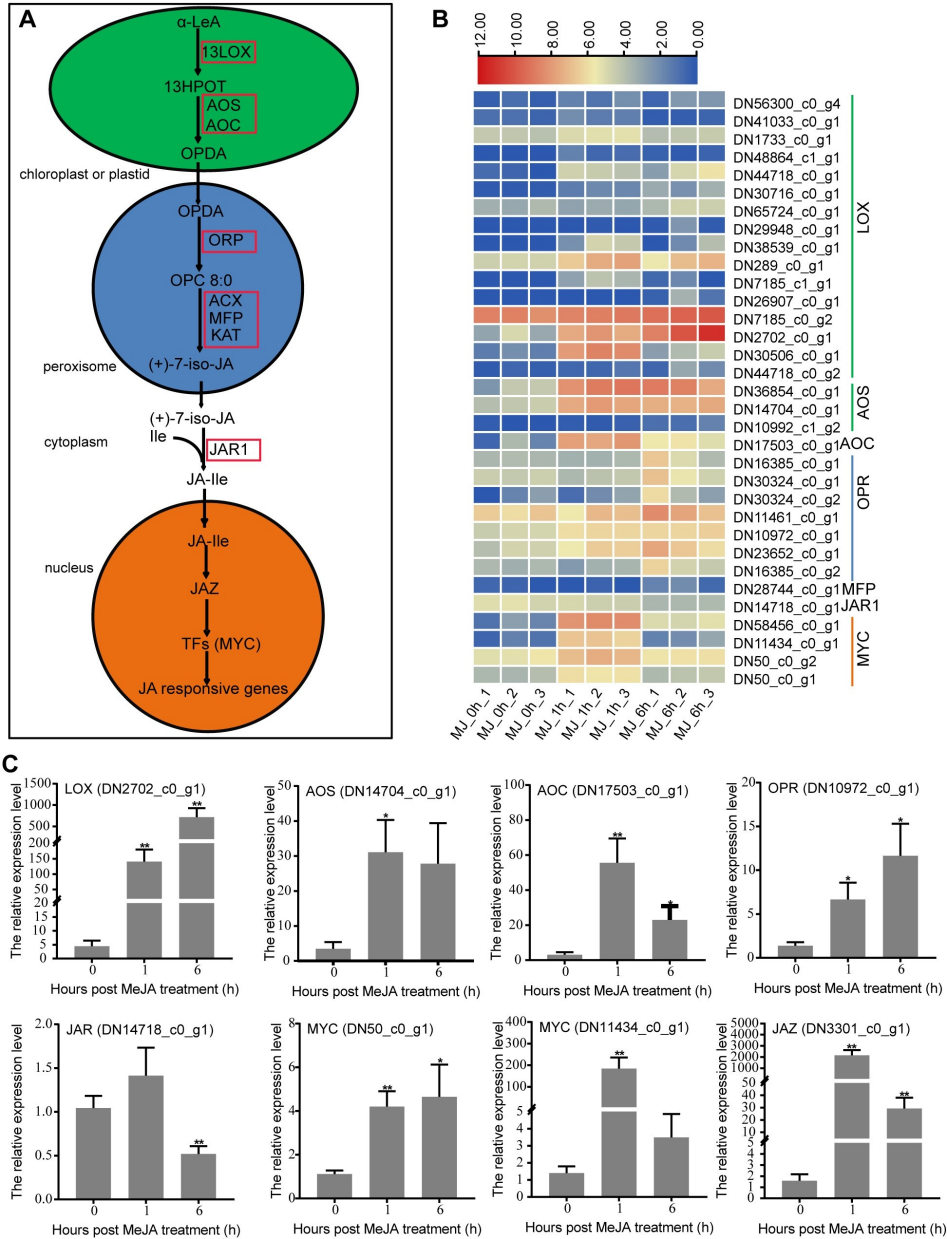
Analysis of DEGs related to JA biosynthesis and signal transduction in *C*. *wenyujin* with MeJA-treatment. (A) JA biosynthesis and signal transduction pathway. Genes with red box represent enzyme genes in this pathway (B) Transcriptional profiles of DEGs related to JA biosynthesis and signal transduction pathway. The log2 (TPM) values for the DEGs were calculated based on three biological replicates for each time-point. The color scale from blue to red represents increase expression level. (C) Expression level of DEGs determined by qRT-PCR. Three independent biological replicates were performed. Asterisks indicate significant difference (*P < 0.05; **P < 0.01).

Twenty-nine DEGs involved in JA biosynthesis were identified (Fig 6A and 6B, and S4 Table). For JA biosynthesis, the initial substrate α-linolenic acid (18:3) (α-LeA) is converted to (9S,13S)-12-oxo-phytodienoic acid (OPDA) catalyzed by 13-lipoxygenase (LOX), allene oxide synthase (AOS), and allene oxide cyclase (AOC) in plastids (chloroplasts). The expression levels of all identified enzyme genes including fourteen *LOX*, three *AOS*, and one *AOC* were up-regulated with MeJA treatment in *C*. *wenyujin* leaves. Subsequently, OPDA is transferred from the chloroplast to the peroxisome and transformed into (+)-7-iso-JA by OPDA reductase 3 (OPR3) and three β-oxidation enzymes: acyl-CoA oxidase (ACX), multifunctional protein (MFP), and L-3-ketoacyl CoA thiolase (KAT). The transcript levels of one *MFP* and seven *OPR* enzyme genes were higher in MeJA-treated *C*. *wenyujin* than those of the untreated samples. Next, (+)-7-iso-JA is transported to the cytoplasm, where it is conjugated with isoleucine (Ile) to form (+)-7-iso-JA-Ile catalyzed by JA-Ile synthetase (JAR1). The *JAR* gene was down-regulated in response to the MeJA treatment in *C*. *wenyujin*. Finally, JA-Ile is transported into the nucleus to activate the JA signaling response.

JAZs are the core factors in JA signaling pathway. Ten *JAZ* genes were up-regulated with MeJA treatment excepting one down-regulated *JAZ* (S5 Table). Under the condition of abundance JA-Ile, JAZs were degraded by ubiquitin (Ub)/26S proteasome pathway, resulting in the release of transcription factors inhibited by JAZs to activate the expression of related downstream genes. Moreover, MYC2 regulates JA-mediated biosynthesis of secondary metabolites as “master switches” [35]. In *C*. *wenyujin*, four putative MYCs were also significantly differentially expressed with MeJA treatment. The relative expression levels of randomly selected genes in JA biosynthesis and signal transduction pathway, were further analyzed by qRT-PCR to verify the reliability of RNA-seq data. The results of expression trends were consistent with the RNA-seq data. The up-regulated fold changes determined by qRT-PCR were higher than that in RNA-seq data (Fig 6C). In addition, several DEGs were identified to be key factors in response to other plant hormones, including gibberellin (GA), auxin, abscisic acid (ABA), and ethylene (ET) (S5 Table). This result suggested that there might be signal crosstalk between the JA signal transduction and other hormone signals.

### Identification and analysis of CwTPSs induced by MeJA in *C*. *wenyujin*

**Fig 7:**
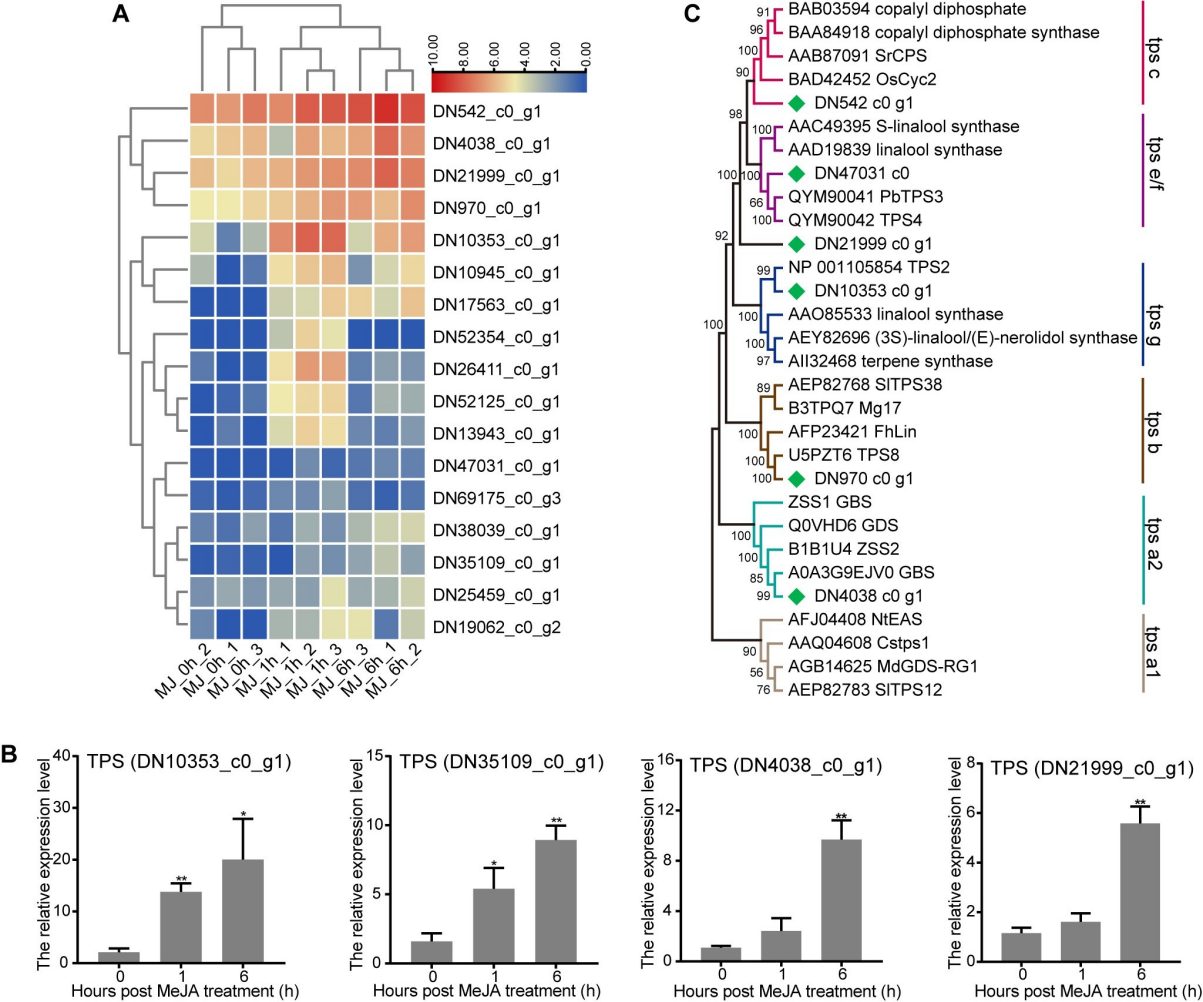
Analysis of CwTPSs induced by MeJA in *C*. *wenyujin*. (A) Transcriptional profiles of identified *CwTPSs*. The log2 (TPM) values for the DEGs were calculated based on three biological replicates for each time-point. The color scale from blue to red represents increase expression level. (B) Phylogenetic tree of CwTPSs with other TPSs from different plants. (C) Expression level of randomly selected *CwTPSs* determined by qRT-PCR. Three independent biological replicates were performed. Asterisks indicate significant difference (*p < 0.05; **p < 0.01).

To identify the TPSs induced by MeJA in *C*. *wenyujin*, unigenes containing the “Terpene_synth” domain (PF01397, PF03936, or PF19086) were screened from all DEGs. Then, 17 DEGs encoding putative TPSs were acquired, and their deduced amino acid sequences were listed in S6 Table. The expression levels of all 17 *TPS* genes were increased with 1 h or 6 h MeJA treatment and partially validated by qRT-PCR (Fig 7A and 7B). The putative TPSs with less than 400 amino acid residues were considered to be partial and discarded. Eventually, only six TPSs were selected for further analysis. The size of the predicted protein sequences ranged from 518 to 811 amino acids. Unigene DN970_c0_g1 showed 90% identity with terpene synthase gene *HcTPS8* from *Hedychium coronarium*. HcTPS8, located in plastids, catalyzed the formation of linalool from GPP and converted FPP to α-bergamotene, cis-α-bisabolene, β-farnesene and other ten sesquiterpenes [36]. DN4038_c0_g1 shared a high similarity (92.15%) with the germacrene B synthase gene from *Curcuma zedoaria* [37]. DN21999_c0_g1 and DN542_c0_g1 were probable diene synthase genes, because of high similarities (> 94%) with putative abietadiene synthase and levopimaradiene synthase genes from *Z*. *officinale*, respectively. DN47031_c0_g1 showed 91.35% identity with a putative S-linalool synthase-like gene from *Z*.*officinale*. DN10353_c0_g1 showed 89.86% identity with a predicted (3S,6E)-nerolidol synthase 1-like gene from *Zingiber officinale*. Nerolidol is an important sesquiterpene alcohol in plants with multi-faceted pharmacological and biological activities [38].

Phylogenetic tree was constructed using these identified unigenes and biochemically characterized TPSs from other plants (Fig 7C). TPSs from other plants used in phylogenetic analysis were listed in S7 Table. DN4038_c0_g1 was classified into TPSa2 subfamily for sesquiterpene synthesis. Unigene DN970_c0_g1 phylogenetically gathered together with known HcTPS8 in TPSb for monoterpene biosynthesis. DN542_c0_g1 might be a diterpene synthetase gene in TPSc subfamily. DN21999_c0_g1 was not classified into any subfamily but close to DN542_c0_g1 in evolutionary relationship. DN10353_c0_g1 and DN47031_c0_g1 belonged to TPSg and TPSe/f subfamilies, respectively, which were summarized for monoterpene, sesquiterpene, and diterpene biosynthesis.

### Putative transcriptional regulation mechanism of CwTPSs

**Fig 8:**
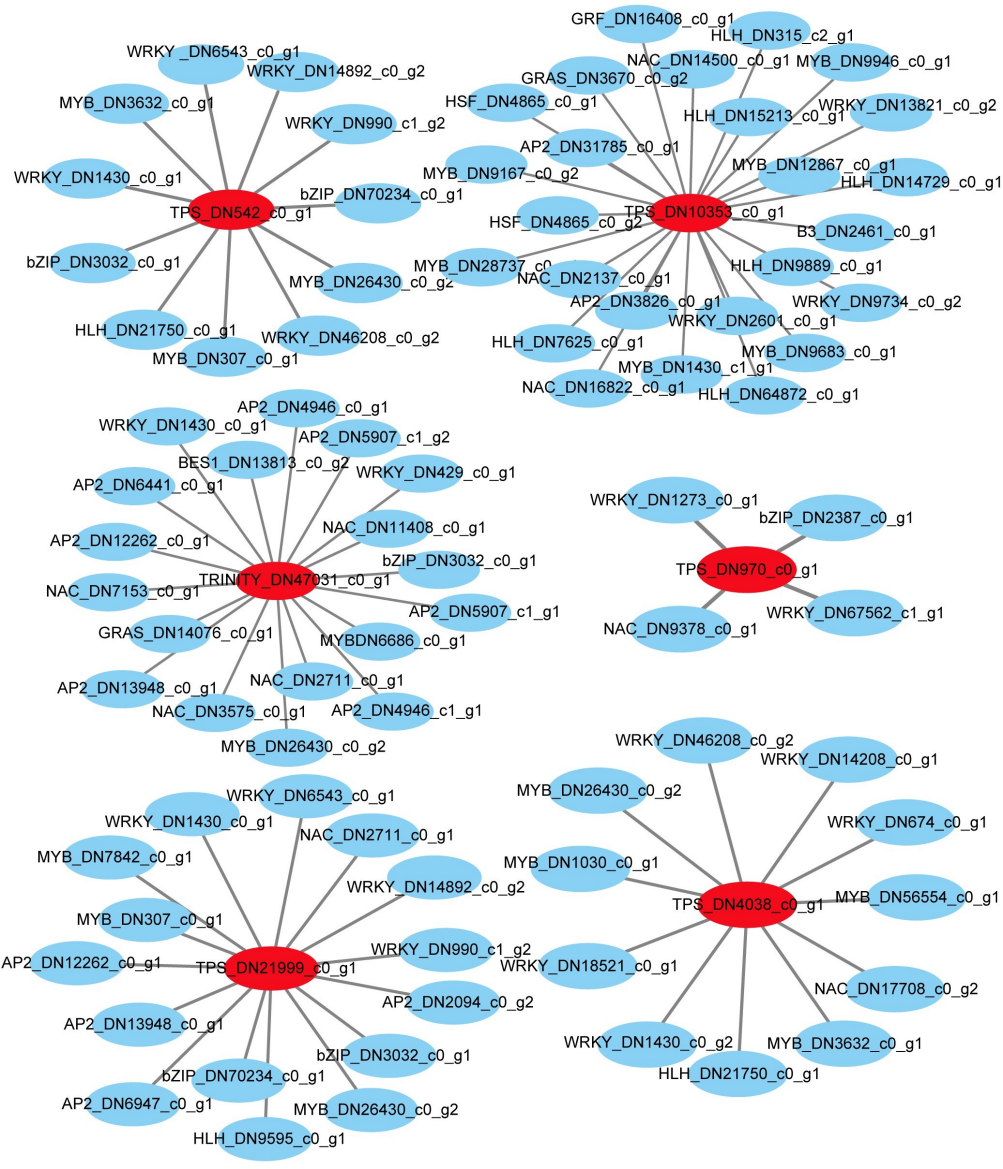
Co-expression network of differentially expressed *CwTPSs* and TF genes in *C*. *wenyujin* with MeJA-treatment. Each node represents a gene with its serial number and annotation information. Red nodes are *CwTPSs*, and blue nodes are TFs. Connecting lines represent co-expression relationships.

JA-responsive TFs have been reported to regulate the JA-induced accumulation of secondary metabolites in plants [39]. In this work, a total of 482 DEGs were annotated in the plant transcription factor database (PlantTFDB, http://planttfdb.cbi.pku.edu.cn/). After MeJA induction, the expression levels of 273 and 332 unigenes were significantly regulated in “control vs 1 h” and “control vs 6 h” groups, respectively. Among them, 123 unigenes were detected in both groups (S4A Fig). All these MeJA-induced TFs were classified into 25 families including MYBs (91), AP2/ERFs (56), WRKYs (53), NACs (42), bHLH (39) and others (S4B Fig). To further identify putative regulators of the terpenes biosynthesis pathway, the expression correlation analysis of *CwTPSs* along with all TFs mentioned above, was conducted using spearman method with correlation coefficient threshold > 0.9 and q value < 0.05 (Fig 8, S8 Table). The results showed that *CwTPS* (DN10353_c0_g1) was co-expressed with 25 TFs including *MYB*, *WRKY*, *NAC*, *HLH*, *AP2/ERF* genes et al., while *CwTPS* (DN970_c0_g1) was only co-expressed with 4 TF genes. DN4038_c0_g1 annotated as *GBS* was co-expressed with 11 TFs including *WRKY*, *MYB*, *HLH*, *bZIP*, and *NAC* genes. *CwTPS* (DN542_c0_g1) was co-expressed with 12 TFs, while *CwTPS* (DN47031_c0_g1) was predictively regulated by 18 TFs. *CwTPS* (DN21999_c0_g1) was co-expressed with 16 TF genes such as *MYB*, *NAC*, *bZIP*, *WRKY*, *AP2/ERF*, and *HLH*. The known key TF genes involved in other hormone signaling pathway were also co-expressed with *CwTPSs*, such as PF01397-related *ARF* and *IAA* genes, ABA-related *ABI* gene, ethylene-related *ERF* and GA-related *GRAS* (*DELLA*) genes. The results revealed that terpenes biosynthesis was regulated by JA signal and its hormonal crosstalk with ABA, ethylene, GA and auxin.

## Discussion

The exogenous application of MeJA was developed as a noninvasive method to induce the accumulation of terpenes in plants. For instance, JA up-regualted α-copaene and β-caryophyllene biosynthesis in *S*. *glabra* [17], terpenoid resin components biosynthesis in *Picea abies* [40], artemisinin biosynthesis in *A*. *annua* [41], and tanshinone biosynthesis in *S*. *miltiorrhiza* [42], respectively. However, the understanding of MeJA-mediated regulation of terpene biosynthesis is limited in *C*. *wenyujin*. So the transcriptome profiling of *C*. *wenyujin* leaves induced by MeJA was analyzed in this study. Transcriptome analysis led to the identification of 7,246 unigenes that were differentially expressed in response to MeJA treatment. Dramatic differences in expression were observed with 6 h MeJA treatment, during which the number of DEGs increased near two-fold compared with that after 1 h induction (Fig 1). Gene expression pattern clustering and DEGs functional enrichment revealed that unigenes hidden in profile 6 fastly responded to MeJA signal at early stage and maintained status of activation, while unigenes hidden in profile 4 were involved in terpenes biosynthesis with 6 h MeJA-treatment (Fig 2 and S2 Fig). Further, DEGs were significantly enriched in JA biosynthesis, JA signaling transduction, and terpenes biosynthesis pathways, suggesting that both JA and terpenes related pathways were affected by MeJA treatment in *C*. *wenyujin* (Figs 3 and 4). One major regulatory mechanism of MeJA-mediated secondary metabolite production in many plants, is to control related genes in biosynthesis pathway [43, 44]. Consistent with this regulatory mechanism, related genes in JA biosynthesis, JA signaling transduction, and terpenes biosynthesis pathway in *C*. *wenyujin* with MeJA treatment were significantly differentially expressed (Figs 5 and 6).

### Regulation of terpenes biosynthesis in MeJA-treated *C*. *wenyujin*

The β-elemene content, the main active ingredient of *C*.*wenyujin*, was significantly increased after MeJA treatment (Fig 5). MeJA application led to different responses of genes involved in terpenes biosynthesis pathways. In the MVA pathway, most genes exhibited no difference in expression level with MeJA treatment, excepting that *AACT*, *HMGS*, and two rate-limiting enzymes *HMGR* genes exhibited increased expression levels. However, most genes in the MEP pathway, such as *DXS*, *DXR*, *HDS*, and *HDR*, were dramatically up-regulated (Fig 5). Similarly, the vast majority of genes related to the patchoulol biosynthesis (a sesquiterpene compound) were up-regulated in *P*. *cablin* after MeJA treatment [18]. In contrast, only *HMGR1* and *HMGR2* in the MVA pathway exhibited increased expression levels, whereas, the majority of genes in the MEP pathways were dramatically down-regulated in *S*. *glabra* with MeJA treatment [17]. Therefore, the response of structure genes of terpene backbone biosynthesis pathway to MeJA elicitation is diverse in different plants. We speculate that the different response patterns are related to the cross-talk between MVA and MEP pathway [6]. In addition, seventeen *TPS* genes encoding terpene synthases showed significantly differential expression with MeJA treatment in *C*. *wenyujin* (Fig 7). Terpene synthase (TPS) is the key enzyme that directly catalyzes the production of various terpenes. All the above DEGs were directly involved in MeJA-induced terpene biosynthesis. In summary, it was apparent that MeJA activated the expression of genes related to terpene biosynthesis pathway in *C*. *wenyujin*. However, the specific regulation mechanisms still need further investigation.

### Regulation of JA biosynthesis and signal transduction in MeJA-treated *C*. *wenyujin*

Alpha-linolenic acid originating from chloroplast membranes is considered as an important precursor for JA biosynthesis [45]. Undergoing condensation, cyclization, and β-oxidation reactions, JA was ultimately formed by catalysis of LOX, AOS, AOC, OPR, ACX, MFP and KAT enzymes (Fig 6A). In the present study, results of RNA-seq suggested that the expression of putative JA biosynthesis genes (*LOX*, *AOS*, *AOC*, *OPR* and *MFP*) were up-regulated with MeJA treatment, which were confirmed by qRT-PCR (Fig 6B and 6C). The result was similar with exogenous MeJA-induced self-activation of JA biosynthesis in *Hevea brasiliensis* [44]. The increased accumulation of endogenous JA induced by exogenous MeJA further triggers the JA signal transduction. JAZs and MYCs were reported as the core factors of JA signal transduction network [12]. Majority of the putative *JAZs* and *MYCs* were highly expressed with MeJA treatment, suggesting the activation of JA signal transduction network (Fig 6 and S5 Table). Apekshika T. Premathilake et al. also reported that several important JA signaling factors as *COI1*, *JAZ*, and *MYC2* were differentially expressed after MeJA induction [16].

Besides, several DEGs were revealed to be involved in signal transduction pathways related to other hormones, including GA, auxin, ABA, and ethylene (S5 Table). For example, ethylene response factor ERF1 and ethylene insensitive factor EIN3 required for JA-ET signal interaction [46, 47], the transcripts of ABA receptor PYR/PYL proteins linked to ABA-JA crosstalk [48], ARF and IAA proteins related to JA-auxin crosstalk [49], DELLA protein related to JA-GA cross-talk [50], were differentially expressed after MeJA induction. It seemed that exogenous MeJA elicitor caused an extensive cascade reaction in plants and ultimately mediated transcription regulation of secondary metabolic pathways. It remains to be validated in future studies for hormones to participate in regulation of terpene biosynthesis by crosstalk in *C*. *wenyujin*.

### Transcriptional regulation of MeJA-mediated terpene biosynthesis in *C*. *wenyujin*

Transcription factors, including the MYB, NAC, WRKY, and HLH families et al., have been reported to be induced by MeJA and involved in secondary metabolite biosynthesis [16, 19, 51]. In this study, transcriptome analysis revealed that 482 putative TFs belonging to 25 TF families were induced by MeJA in *C*. *wenyujin* (S4 Fig). In particular, *MYBs* exhibited significantly differential expression in responding to MeJA induction. The expression patterns of *MYB* genes were correlated with those of the five *CwTPSs*, suggesting that *MYBs* may serve as important regulators of terpene biosynthesis in *C*. *wenyujin* (Fig 8). In the flowers of *Freesia hybrida* and *A*. *thaliana*, MYB21 interacted with MYC2 to activate the expression of *TPS* genes [52]. Not coincidentally, HcMYB2 activated the expression of linalool synthase gene *HcTPS5* to regulate the biosynthesis of floral aroma compounds in *Hedychium coronarium* [53]. In addition, *WRKY* genes were highly correlated with *TPS* genes in responding to MeJA in other plants [17, 54]. Consistent with these results, *WRKY* unigenes were also highly correlated with six deduced *CwTPS* unigenes in *C*. *wenyujin* in this study. Although transcriptome comparative analysis can establish the relationship between TFs and metabolites biosynthesis pathway, it is not enough to reveal the detail regulatory mechanism. The molecular mechanisms between TFs and function genes are different and diverse in different plants, which still remain elusive. Additional research is needed to clarify how these candidate TFs regulate the terpene biosynthesis, and whether this regulation is dependent or independent of JA signaling in *C*. *wenyujin*.

## Conclusions

Our RNA-seq analysis of MeJA-treated *C*. *wenyujin* indicated that numerous unigenes were transcriptionally regulated, including genes related to JA biosynthesis, JA signal transduction, and terpenes biosynthesis. Meanwhile, TFs, which may be implicated in regulation of terpene biosynthesis, were also induced by MeJA. In treated *C*. *wenyujin*, MeJA triggered the expression of genes involved in endogenous JA biosynthesis (*LOX*, *AOS*, *AOC*, *OPR*, *MFP*, and *JAR*) and JA signal transduction (*MYC2* and *JAZ*). Then through JA signal transduction network, TFs showed significant response to MeJA treatment, resulting in increased expression of key genes related to terpenes biosynthesis. These results provide new insights into the molecular regulation mechanisms of terpene biosynthesis, which are also helpful to identify candidate genes for regulating the accumulation of valuable compounds in *C*. *wenyujin*.

## Supporting information

S1 Fig DEGs in MeJA-treated *C*. *wenyujin*. The red, green, and gray dots show up-regulated, down-regulated, and no change expression level of DEGs, respectively. Strict screening conditions as following: 1) the expression levels were up or down 2-folds regulation, i.e. |log2Fold Chang| ≥ 1; 2) *P* value < 0.05.
(TIF)

S2 Fig GO enrichment analysis of genes from profile 4 and profile 6 in *C*. *wenyujin* with MeJA treatment. The Y-axis on the right side represents the GO terms and X-axis represents rich factor. The size of the dot indicates the number of genes in this GO term, and the color of the dot corresponds to different FDR (*p*-vaule). *P*-vaule < 0.05 indicates significant enrichment of GO function. Top 20 of significant enrichment results are displayed.
(TIF)

S3 Fig GO classification of all DEGs in *C*. *wenyujin* with MeJA treatment. (TIF)

S4 Fig Statistics and classification of differentially expressed transcription factors in *C*. *wenyujin* with MeJA treatment. (TIF)

S1 Table Primers for qRT-PCR. (XLSX)

S2 Table Detailed statistics of the RNA-seq data. (XLSX)

S3 Table Differentially expressed genes involved in terpenoid backbone biosynthesis. (XLSX)

S4 Table Differentially expressed genes involved in JA biosynthesis. (XLSX)

S5 Table Differentially expressed genes involved in plant hormone signal transduction. (XLSX)

S6 Table Differentially expressed TPS genes in *C*. *wenyujin*. (XLSX)

S7 Table TPSs from other plants used in phylogenetic analysis. (XLSX)

S8 Table Expression correlation of differentially expressed *CwTPSs* and TFs in *C*. *wenyujin* with MeJA-treatment. (XLSX)
