# Supplementary figures and images for: Transcriptome analysis reveals regulation mechanism of methyl jasmonate-induced terpenes biosynthesis in Curcuma wenyujin
Source: PLoS One. 2022 Jun 23;17(6):e0270309. doi: 10.1371/journal.pone.0270309 (PMC9223393; doi:10.1371/journal.pone.0270309)

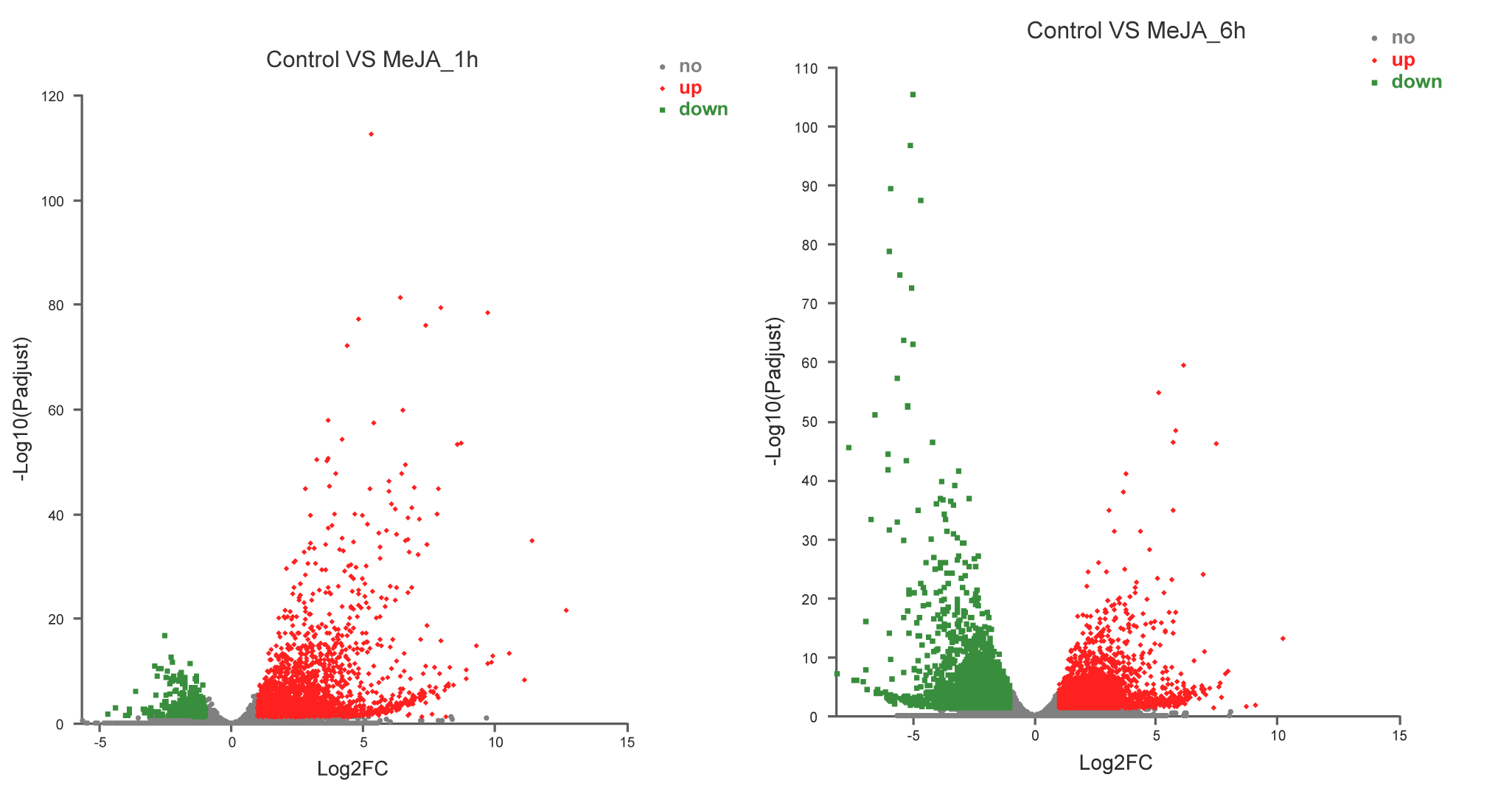

Supplement: S1 Fig — The red, green, and gray dots show up-regulated, down-regulated, and no change expression level of DEGs, respectively. Strict screening conditions as following: 1) the expression levels were up or down 2-folds regulation, i.e. |log2Fold Chang| ≥ 1; 2) P value < 0.05. (TIF) [file pone.0270309.s001.tif]

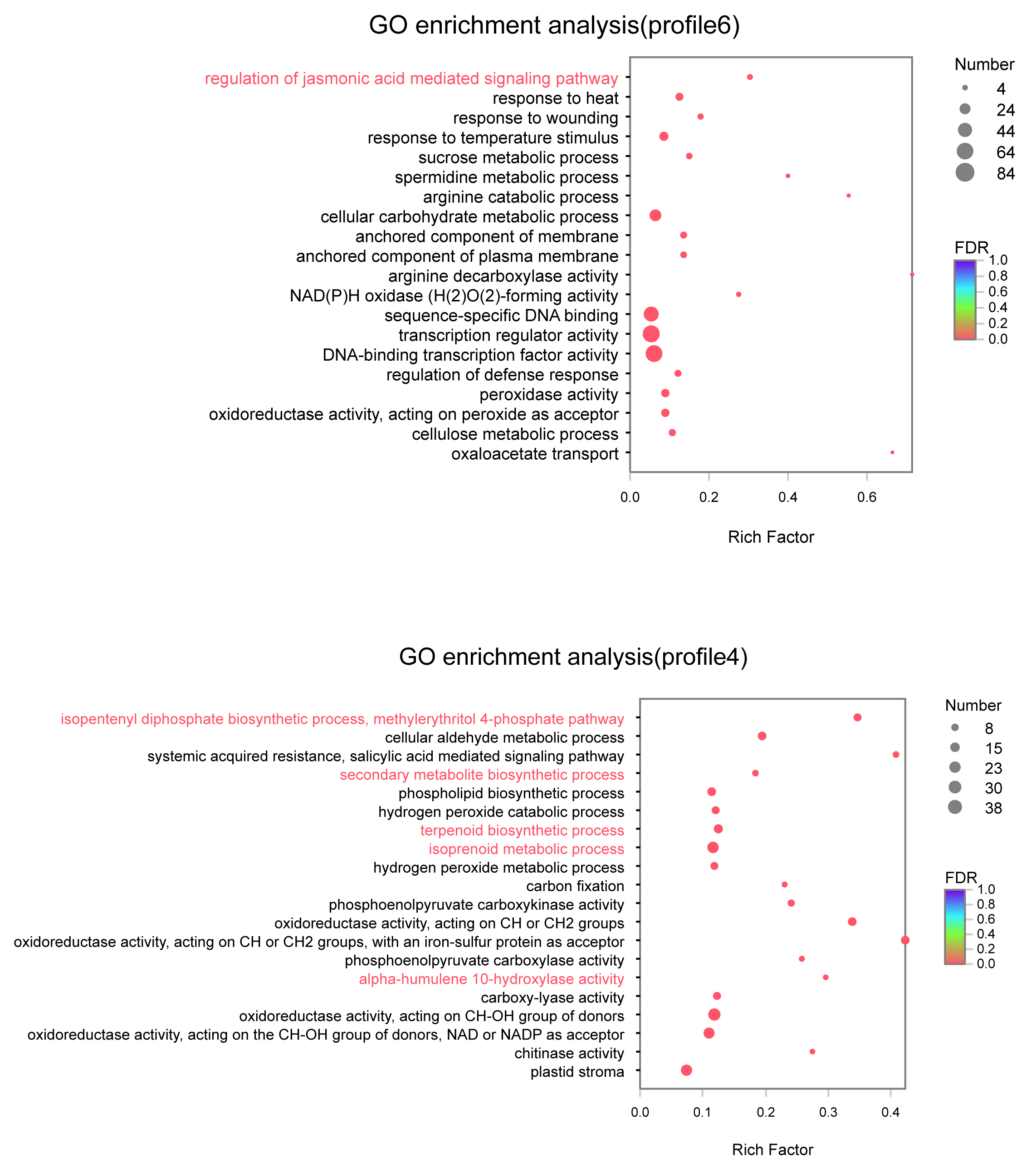

Supplement: S2 Fig — The Y-axis on the right side represents the GO terms and X-axis represents rich factor. The size of the dot indicates the number of genes in this GO term, and the color of the dot corresponds to different FDR (p-vaule). P-vaule < 0.05 indicates significant enrichment of GO function. Top 20 of significant enrichment results are displayed. (TIF) [file pone.0270309.s002.tif]

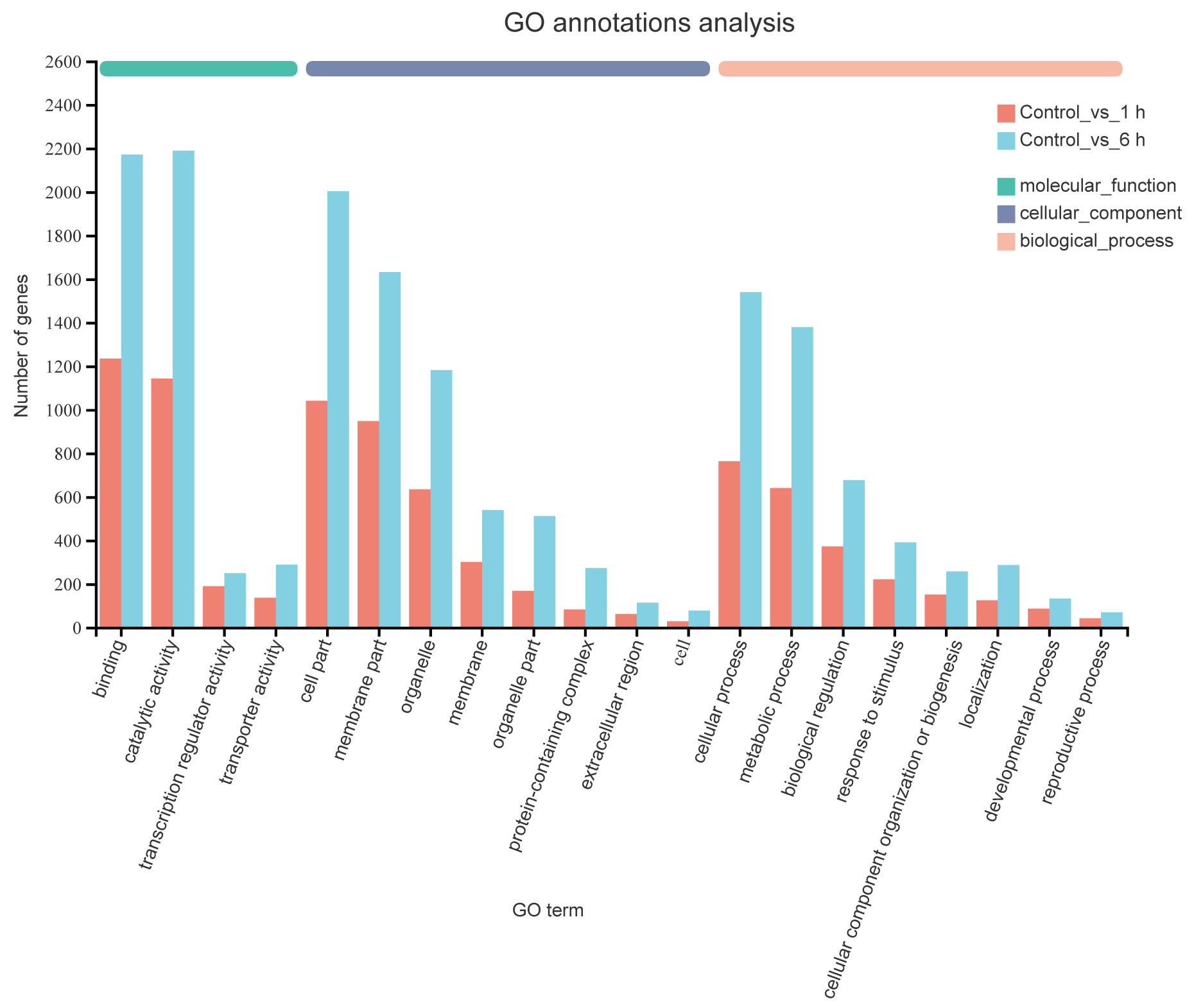

Supplement: S3 Fig — (TIF) [file pone.0270309.s003.tif]

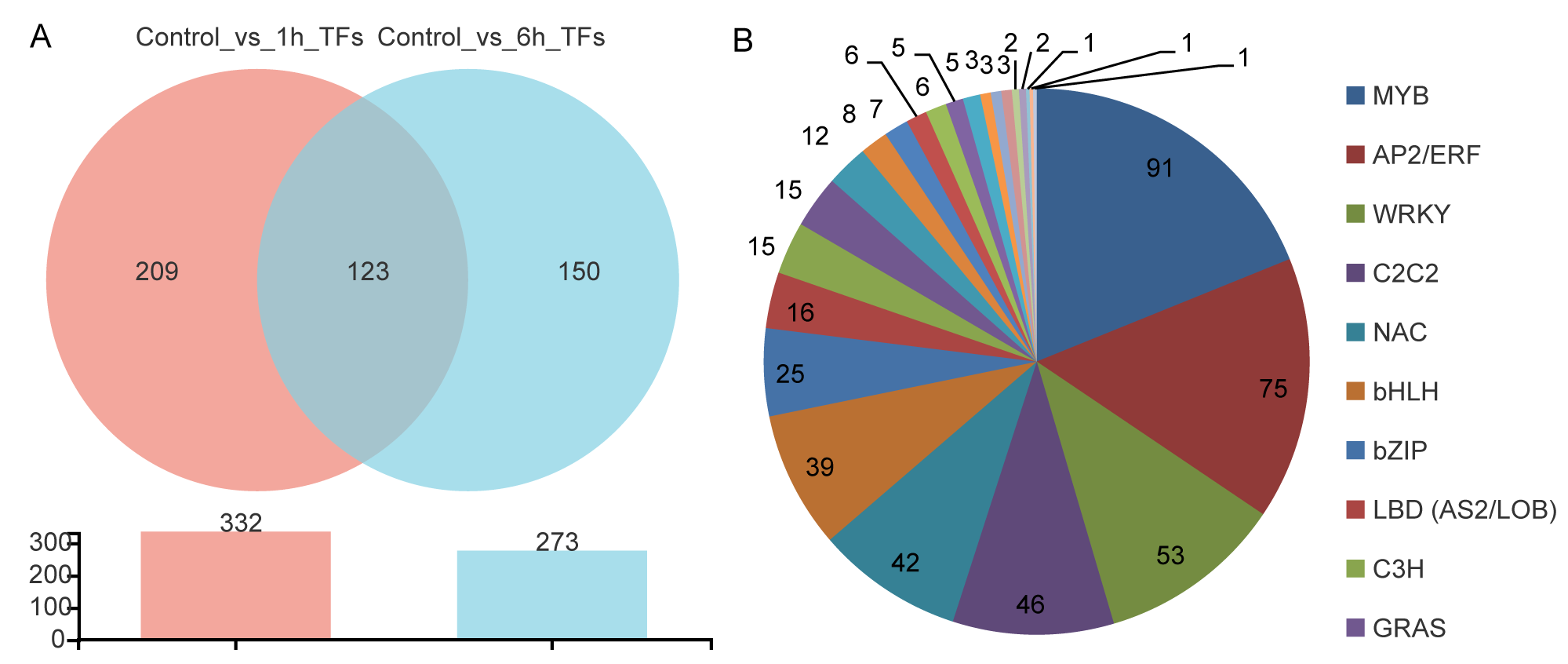

Supplement: S4 Fig — (TIF) [file pone.0270309.s004.tif]
